# Region-specific differential corneal and scleral mRNA expressions of *MMP2*, *TIMP2*, and *TGFB2* in highly myopic-astigmatic chicks

**DOI:** 10.1038/s41598-017-08765-6

**Published:** 2017-09-12

**Authors:** Lisa Yan-yan Xi, Shea Ping Yip, Sze Wan Shan, Jody Summers-Rada, Chea-su Kee

**Affiliations:** 1 School of Optometry, The Hong Kong Polytechnic University, Hung Hom, Kowloon, Hong Kong SAR; 2 Department of Health Technology and Informatics, The Hong Kong Polytechnic University, Hung Hom, Kowloon, Hong Kong SAR; 3 Department of Cell Biology, University of Oklahoma Health Sciences Center, Oklahoma City, OK United States; 4 Interdisciplinary Division of Biomedical Engineering, The Hong Kong Polytechnic University, Hung Hom, Kowloon, Hong Kong SAR

## Abstract

Myopia and astigmatism, two common refractive errors frequently co-exist, are affecting vision at all working distances in the affected populations worldwide. Eyeballs having these refractive errors are known to exhibit abnormal eye shape at the anterior and posterior eye segments, but whether the outer coats of these abnormal eyeballs, cornea anteriorly and sclera posteriorly, are regulated by region-specific molecular mechanism remains unclear. Here we presented the changes in mRNA expression levels of three genes (*MMP2*, *TIMP2*, and *TGFB2*), all known to participate in extracellular matrix organization, at five regions of the cornea and sclera in chickens developing high myopia and astigmatism induced by form deprivation. We found that, compared to normal chicks, the highly myopic-astigmatic chicks had significantly higher expression of all three genes in the superior sclera (Mann-Whitney tests, all p ≤ 0.05), as well as higher *TIMP2* expression in the central cornea and nasal sclera (Mann-Whitney tests, both p ≤ 0.05). Strikingly, the superior scleral region stood out as showing the strongest and most widespread correlations between mRNA expression and biometry parameters including axial and astigmatic components (r = + 0.52~ + 0.85, all p < 0.05). These results imply that local molecular mechanism may manipulate the eye shape remodeling across the globe during refractive-error development.

## Introduction

Myopia, or short-sightedness, is a highly prevalent visual condition associated with significant risks of morbidity. Despite its worldwide public health impact, the etiology of myopia remains poorly understood. Epidemiological studies on geographically diverse populations showed a higher prevalence of myopia in Chinese ethnicity^1^; of particular concern is the increasing trend of highly myopic population^2^. Although the vision of high myopes may be corrected by ophthalmic aids, their larger and longer eyeballs are associated with increased risks of sight-threatening eye diseases (e.g., glaucoma and cataract) and posterior segment anomalies (e.g., retinal detachment, staphyloma and tilted optic disc)^3, 4^.

The external ocular tissues responsible for maintaining the eye’s biomechanical properties – the cornea anteriorly, and the sclera posteriorly – exhibit abnormal structural properties in highly myopic eyes^3, 5–7^. High myopia is frequently associated with an irregular posterior eye shape in humans^5, 8–10^; and in animal models (monkeys^11^; chickens^12^). Importantly, astigmatism, another refractive error due mainly to non-uniform corneal curvature, also frequently co-exists with high myopia (humans^13^, monkeys^14^, and chicks^15^), suggesting abnormal refractive error development may involve structural remodeling of both the anterior and posterior eye segments. While it remains unclear how eye shape is regulated across the globe, there is ample evidence that eye shape remodeling during myopia development involves significant alterations of the components of extracellular matrix (ECM) in humans and animal models^16–22^. However, converging evidence indicates that the structural changes in ametropic eyes may be modulated by mechanisms localized within the eye itself^23^: First, optic-nerve blockade does not prevent the myopia induced by form deprivation or spherical defocus^24–29^. Second, restricting visual manipulations to particular regions of the visual field induces refractive-error and structural changes only in areas corresponding to the deprived retinal regions^12, 30–35^. Whether this local, region-specific mechanism plays a role in the development of myopic-astigmatic error is unclear. One working hypothesis is that the astigmatism associated with high myopia may be a byproduct of asynchronous and/or region-specific structural remodeling during myopia development. We tested this hypothesis by comparing the mRNA expression of 3 target genes (matrix metalloproteinase-2, *MMP2*; tissue inhibitor of metalloproteinase-2, *TIMP2*; transforming growth factor-beta 2, *TGFB2*), all known to participate in scleral structural remodeling (summarized in Supplementary Table S1). Transforming growth factor β (TGF-β) is a multi-functional factor that regulates cells growth and differentiation. TGF-β has been associated with myopia development. With functions in the proliferation regulation of scleral fibroblast cells and ECM production, *TGFB2* plays an important role in biological activities^36^. However, the expression patterns of *TGFB2* are controversial. For example, *TGFB2* protein is upregulated in the posterior sclera of myopic chicks^37–39^, whereas *TGFB2* mRNA expression decreased after form deprivation in tree shrew sclera^39^. Matrix-metalloproteinases (MMPs) are some of the enzymes that degrade ECM molecules. *MMP2* is capable of degrading a range of components of the scleral ECM including collagens and proteoglycans^40^. During myopia development, upregulated *MMP2* expression and increased activity are reported in myopia models, both mammalian^16, 41^ and avian^42, 43^. *MMP2* can subsequently be regulated by tissue inhibitor of metalloproteinases (TIMPs) such as *TIMP2* ^40^. Balance between *MMP2* and *TIMP2* is critical to normal scleral matrix turnover and subsequently, regulation of eye development. Alteration of TIMP levels has been reported during slowed or increased eye growth in different animal models and human studies^18, 44, 45^. Recent study showed that downregulation of *MMP2* expression levels and increased *TIMP2* levels accompanied the pirenzepine-induced suppression of myopia in guinea pig^44^.

The experiments in this study were carried out using the chick form-deprivation paradigm, since large amounts of high myopia and astigmatism are reliably induced using this model, and general (non-region-specific) structural and molecular changes have been widely documented^15, 22, 23, 35^.

## Results

### Effects of form deprivation on refractive and axial components

**Figure 1:**
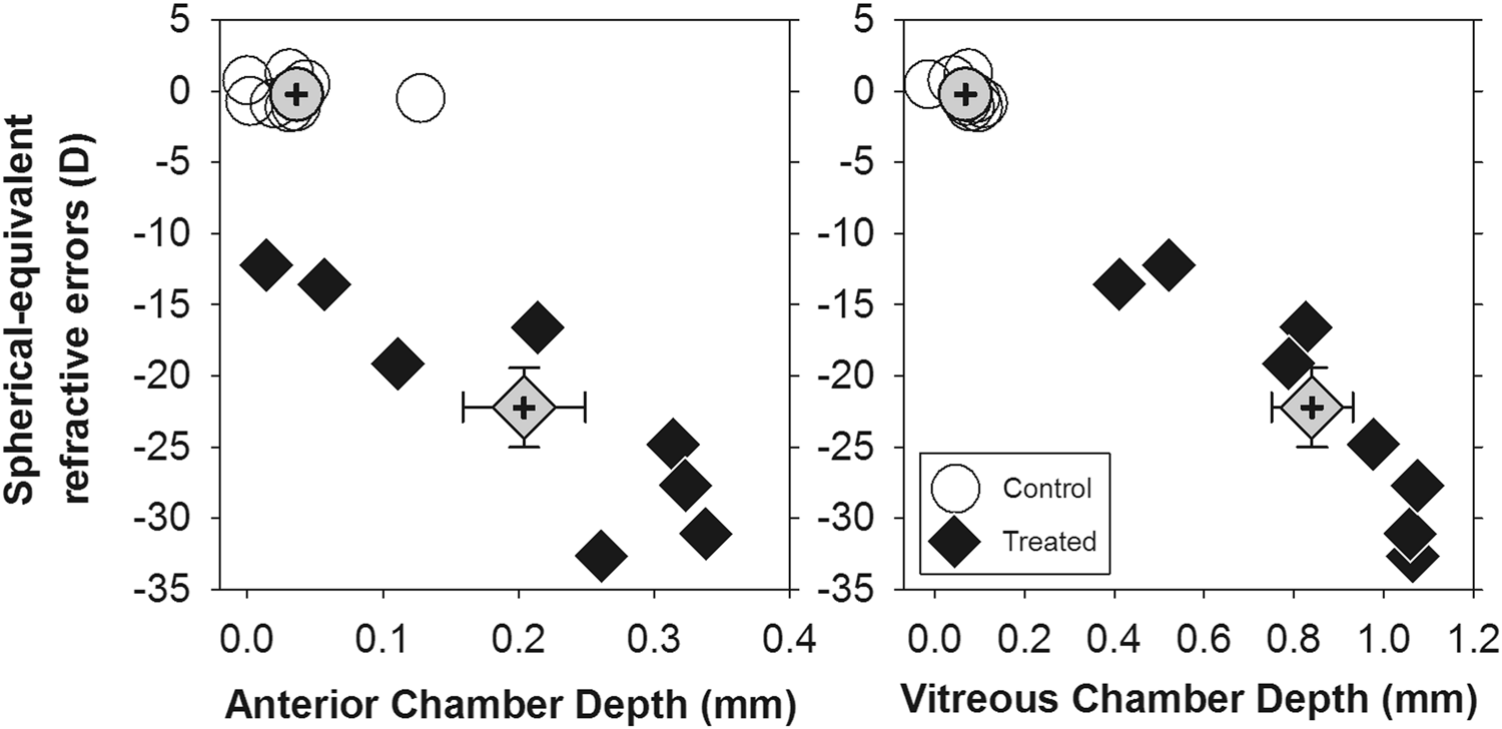
Effects of form deprivation on refractive and axial parameters. Spherical-equivalent refractive errors are plotted against the anterior chamber (left) and vitreous chamber depths (right) at the end of the one-week treatment period of monocular form deprivation. All data are expressed as inter-ocular differences (treated/right eye – fellow/left eye). The data for individual birds are represented by open (normal) or filled (treated) symbols. The mean ± SEM values for each group are represented with grey symbols with error bars, note that the error bars for normal group are masked by the filled symbols.

**Figure 2:**
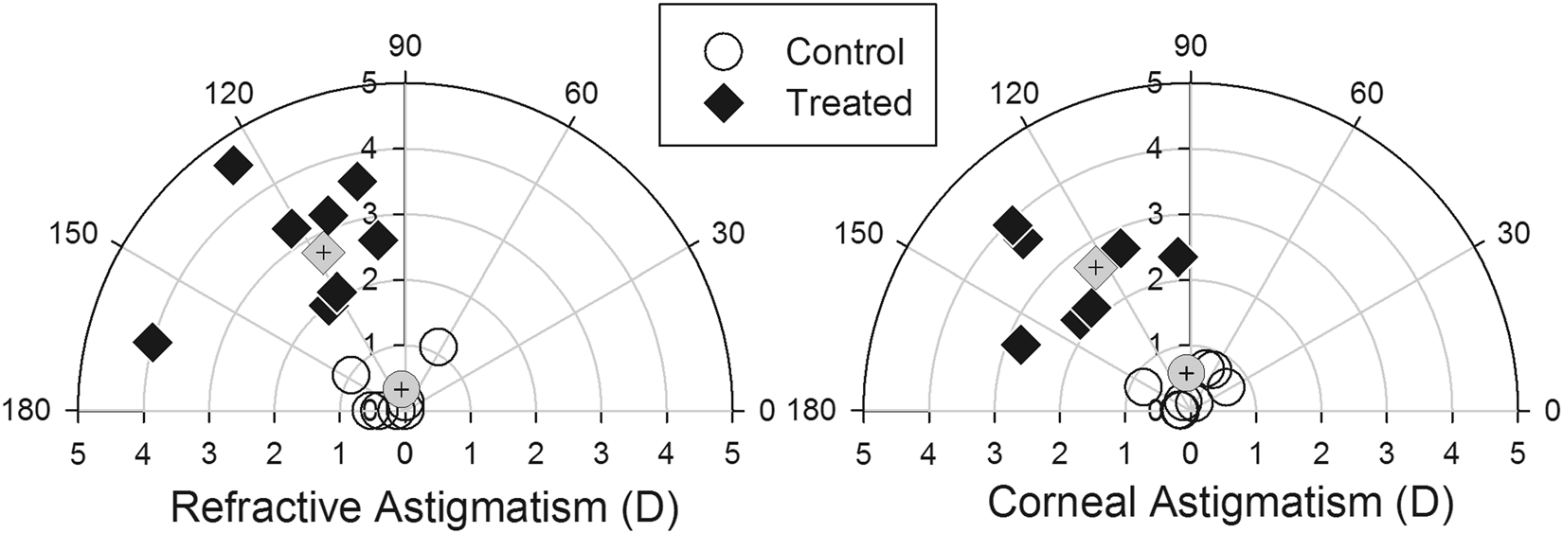
Effects of form deprivation on astigmatism. Refractive (left) and corneal (right) astigmatism at the end of the treatment period for individual normal (open circles) and treated birds (filled diamonds). The magnitude and axis of astigmatism are represented by the distance and angle from the origin, respectively. Average values calculated by power vector analyses are represented by grey symbols with a cross.

In the treated group, the form-deprived eyes developed significantly higher myopia (mean ± SEM: −21.56 ± 2.78D vs. –0.66 ± 0.21D, p < 0.001), deeper anterior chamber depth (mean ± SEM: 1.65 ± 0.05 mm vs. 1.45 ± 0.02 mm, p < 0.01), and longer vitreous chamber depth (mean ± SEM: 6.12 ± 0.10 mm vs. 5.28 ± 0.07 mm, p < 0.001) than the fellow untreated eyes. As expected, the inter-ocular differences (treated/right eye– fellow/left eye) of these parameters were all significantly larger (independent *t*-tests, all p < 0.01) in the treated group than the normal group (mean±SEM: spherical equivalent = −22.22 ± 7.93D vs. −0.25 ± 0.93D; anterior chamber depth = 0.20 ± 0.05 mm vs. 0.04 ± 0.01 mm; vitreous chamber depth = 0.84 ± 0.26 mm vs. 0.07 ± 0.04 mm). Figure 1 illustrates the distributions of the inter-ocular differences in spherical-equivalent refractive errors versus anterior chamber depths (left) and vitreous chamber depths (right) for the normal (open symbols) and treated birds (filled symbols) at the end of the 7-day treatment period. In addition to the myopic errors, the treated chicks also developed significantly higher inter-ocular differences in refractive astigmatism, (mean ± SEM: 3.17 ± 0.32D vs. 0.47 ± 0.14D, p < 0.001) corneal astigmatism (mean ± SEM: 2.74 ± 0.24D vs. 0.43 ± 0.11D, p < 0.001), and J45 astigmatic components (mean ± SEM: refractive: −1.09 ± 0.19D vs. −0.00 ± 0.09D; corneal: −1.14 ± 0.19D vs. 0.06 ± 0.08D; both p < 0.001) when compared to normal birds. The polar plots in Fig. 2 illustrate the distributions of inter-ocular differences in refractive (left) and corneal astigmatism (right) for normal (open) and treated (filled) chicks: The magnitude and axis of the astigmatism for each bird are represented by the distance and angle from the origin respectively. As shown, the refractive astigmatism and corneal astigmatism in the treated birds were much higher than those in normal birds and the axes were oriented slightly obliquely from 90 axis. In addition, correlation analyses showed that refractive, corneal, and ocular axial components were interrelated (Table 1).

**Table 1:** Correlations between refractive parameters and ocular dimensions.

	Refraction			Corneal Topography			A-scan Ultrasonography				
	SE	R.JO	R.J45	CC	C.J0	C.J45	AC	VC	RT	CT	ST
SE											
R.J0											
R.J45	+0.77**										
CC											
C.J0		+0.71**									
C.J45	+0.75**	+0.55*	+0.90**								
AC	-0.75**			+0.74**		−0.54*					
VC	−0.94***		−0.69**	+0.50*		−0.65**	+0.76**				
RT	+0.68**		+0.74**			+0.66**	−0.56*	−0.78***			
CT		−0.51*		−0.50*							
ST										+0.57*	

All data used for analyses were inter-ocular differences. Note that only statistically-significant results are reported.

SE, spherical equivalence; RJ0, refractive J0 astigmatic component, RJ45; refractive J45 astigmatic component; CC, corneal curvature; CJ0, corneal J0 astigmatic component; CJ45, corneal J45 astigmatic component; AC, anterior chamber depth; VC, vitreous chamber depth; RT, retinal thickness; CT, choroidal thickness. *p < 0.05; **p < 0.01; ***p < 0.001.

### Effects of treatment on gene expression

When the inter-ocular differences in gene expressions (ΔΔC_T_) of all five regions from each bird were averaged and compared between groups, the scleral *MMP2* and *TIMP2* expressions in treated group were significantly higher (i.e., lower ΔΔC_T_) than those in normal groups (Mann-Whitney tests, both p < 0.05). No such differences were found in all three genes from the cornea (Mann-Whitney tests, all p > 0.05).

**Figure 3:**
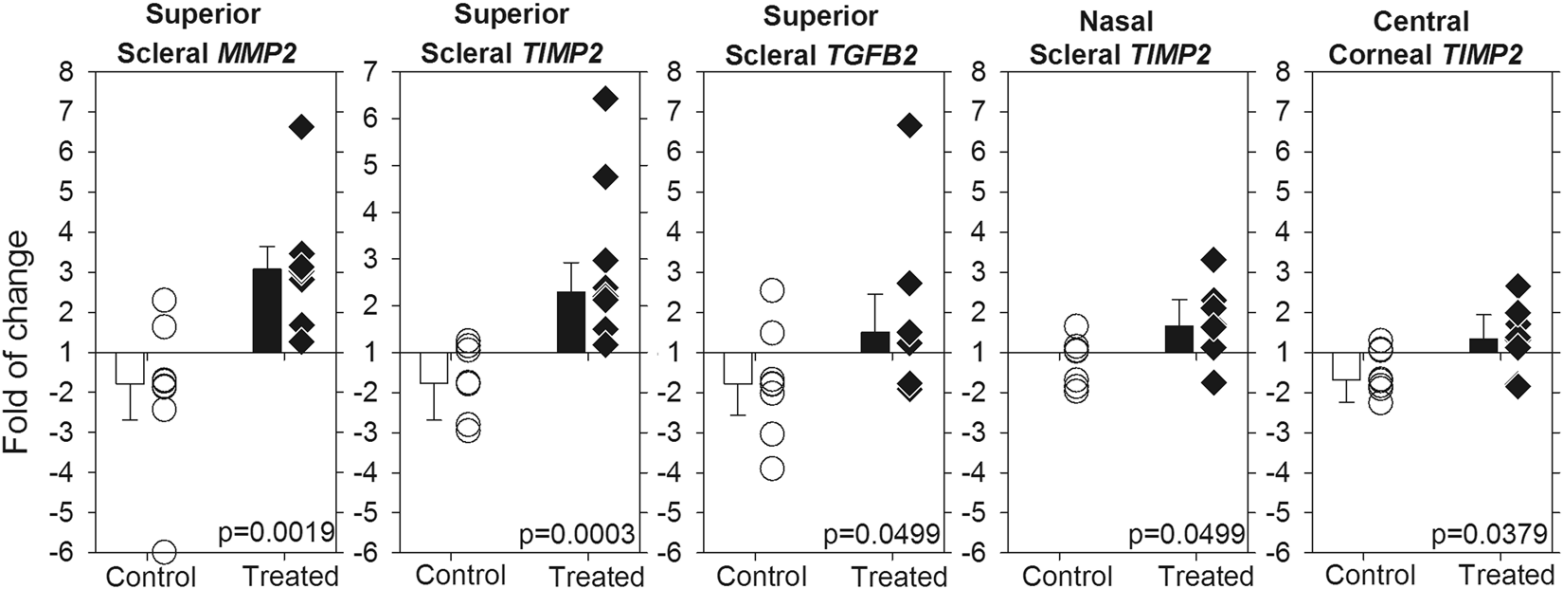
Differences in mRNA expressions at specific regions. Messenger RNA expression differences between the treated group (treated minus fellow untreated; filled symbols) and normal group (right minus left; open symbols) for regions of sclera or cornea that displayed significant between-group differences. Symbols represent individual data and bars represent mean ± SEM for each group. *P* values represent results from Mann-Whitney test.

Analyses of the treatment effects on each gene at individual regions showed that, compared to normal group, the treated group had significantly higher gene expressions in all three genes at the superior sclera (Mann-Whitney tests: *MMP2*, p < 0.01; *TIMP2*, p < 0.001; *TGFB2*, p < 0.05), and in *TIMP2* gene expression in the central cornea and nasal sclera (both p < 0.05). Figure 3 plots the significant fold-change differences in mRNA expressions for individual data and group means.

### Correlation between mRNA expression and ocular component dimensions

**Figure 4:**
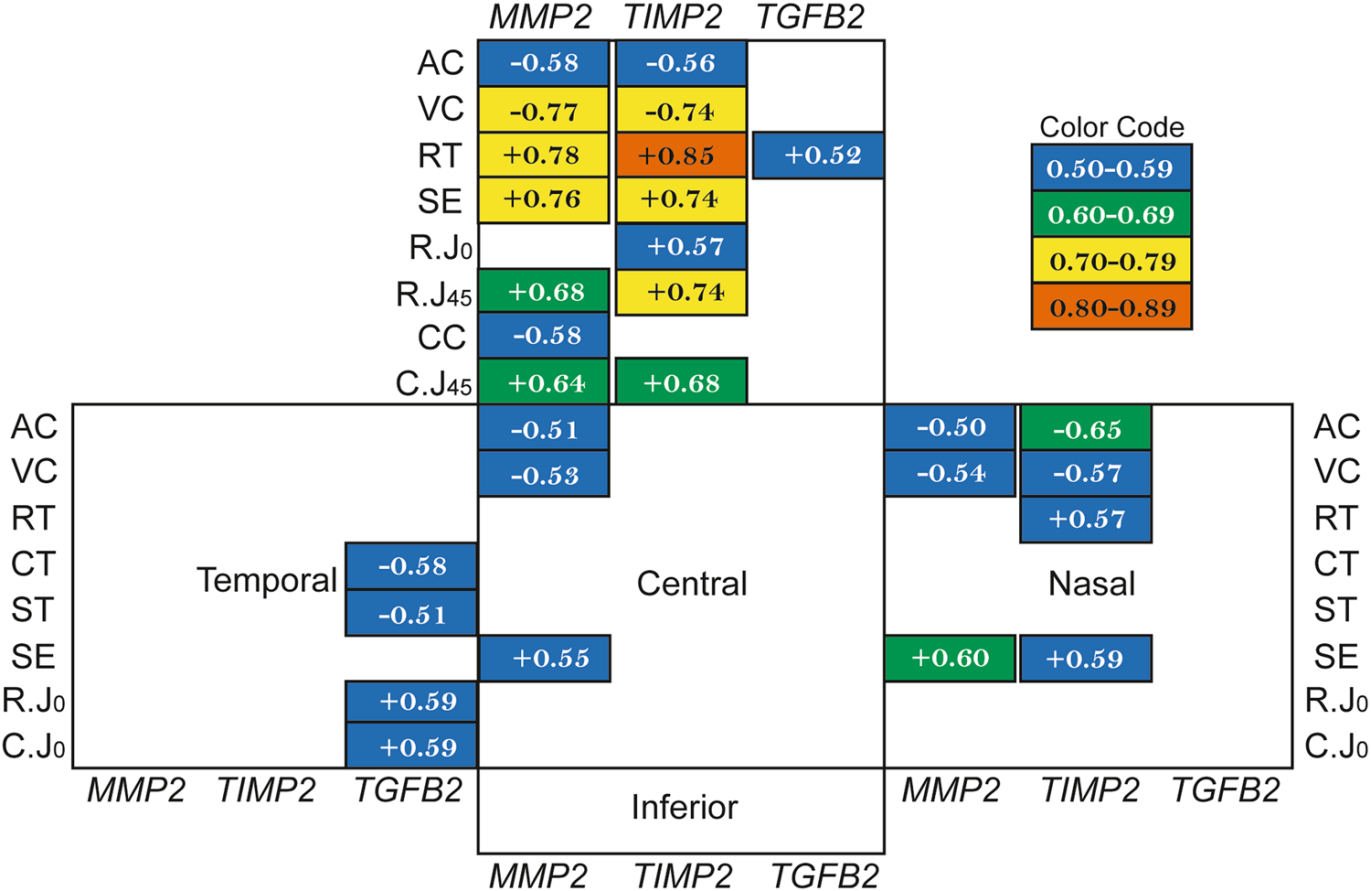
Multiple significant correlations at superior sclera. Pearson’s correlation coefficients (r) between the mRNA expressions (ΔΔC_T_) and multiple ocular parameters at individual scleral regions (boxes). Only statistically significant coefficients are shown, the warmer the color the higher the correlations (see color code). VC, vitreous chamber depth; RT, retinal thickness; AC, anterior chamber depth; ST, scleral thickness;﻿ SE, spherical-equivalent refractive error; R.J45, refractive J45 astigmatic component; CC, average corneal curvature; C.J45, corneal J45 astigmatic component.

To understand if regional mRNA expression level was correlated with refractive error and/or axial ocular component dimensions, we pooled all the data from both treated and normal groups for Pearson’s correlation analyses. Data from both groups were chosen to cover a wide range of changes in refractive errors and ocular components. Fig. 4 shows the statistically significant correlation coefficients between individual biometric components and genes expressions at different scleral regions. The strengths of the correlation are represented using different colors, with warmer colors representing stronger correlations. The superior scleral region stood out as showing the strongest and most widespread correlations between mRNA expression and biometry parameters. Significant correlations were also found at the temporal, nasal and central sclera, but not at the inferior region.

**Figure 5:**
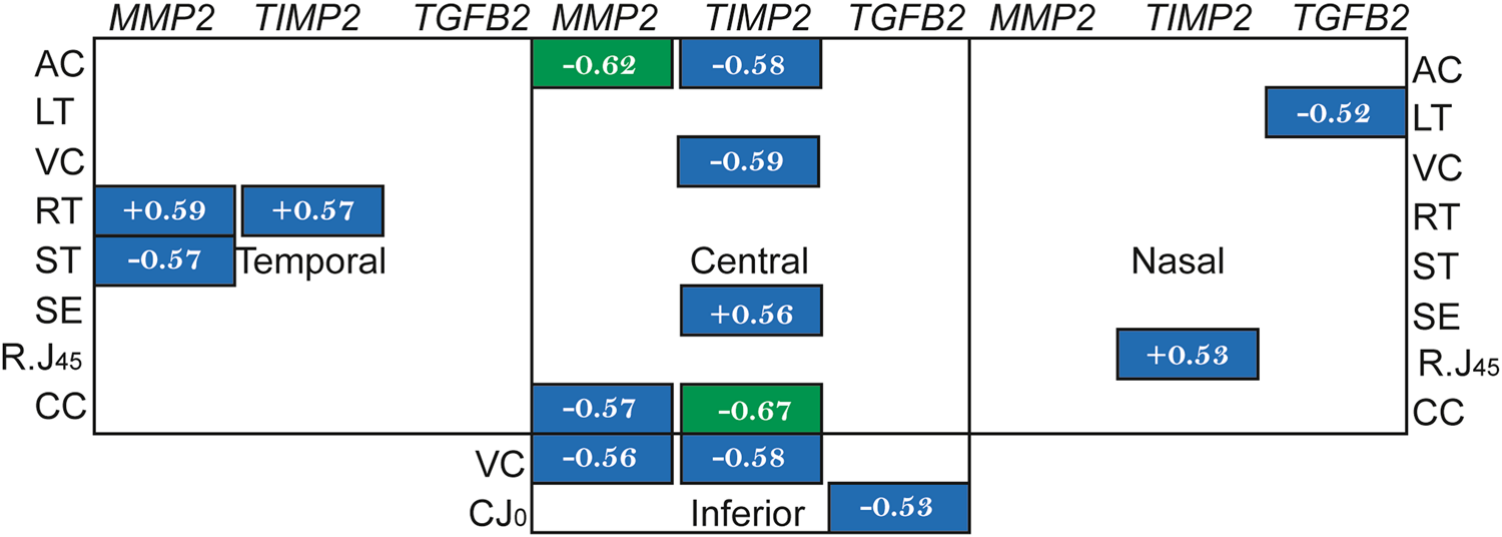
Correlations in cornea. Pearson’s correlation coefficients (r) between the mRNA expressions (ΔΔC_T_) with multiple ocular parameters at four corneal regions. Only statistically significant correlations are shown; green boxes highlight higher correlations than blue boxes (similar color code as in Fig. 4). VC, vitreous chamber depth; RT, retinal thickness; AC, anterior chamber depth; ST, scleral thickness; SE, spherical-equivalent refractive error; CC, average corneal curvature.

In contrast to the sclera, the corneal samples only showed significant correlations between gene expression level and biometry parameters along the horizontal meridian and inferior region (Fig. 5). Here the central cornea showed the highest number of significant correlations. However, even in this region, the correlations were not as high or as numerous as those found in the superior sclera (cf. Fig. 4).

### Correlations of mRNA expressions between genes

**Figure 6:**
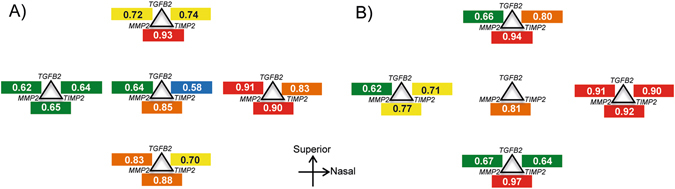
Correlations between pairs of genes. Pearson correlation coefficients of mRNA expression levels (ΔΔC_T_) between pairs of genes at specific scleral (**A**) and corneal (**B**) regions. Only statistically significant coefficients are shown; the warmer the color the higher the correlations (, 0.50~0.59; , 0.60~0.69; , 0.70~0.79; , 0.80~0.89; , 0.90~0.99). All coefficients are positive values.

Figure 6 shows the significant correlations between pairs of genes, in scleral (Fig. 6A) and corneal (Fig. 6B) regions. Only statistically significant Pearson’s coefficients are presented, with warmer colors representing stronger correlations. In both sclera and cornea, significant correlations between *MMP2* and *TIMP2* were found for all five regions. On the other hand, whereas all pairwise correlations were statistically significant across the five scleral regions, significant correlations between all three genes were found in all except the central cornea. Interestingly, the highest correlations between gene expressions were found at the inferior region of cornea (r = 0.97 for *MMP2*-*TIMP2*) and superior region of sclera (r = 0.93 for *MMP2*-*TIMP2*).

## Discussion

Deprivation of form vision has consistently been shown to induce axial myopia in a wide variety of animal models, making this a very common visual manipulation for the study of myopia development. Recent studies from rhesus monkeys^14^ and chicks^15, 35^ showed that form deprivation promoted not only posterior axial elongation but also significant magnitudes of ocular astigmatism, suggesting a mechanistic link between the changes in posterior and anterior eye growth. In the current study, eight of the 39 form-deprived birds exhibited axial elongation and highly myopic-astigmatic errors, the percentage of birds (20.5%) exhibiting myopia <−10D and astigmatism >2D was similar to the percentage (27.2%) from a separate group of birds receiving identical treatment paradigm in a previous study^15^, excluding the possibility that the selected chicks in this study were outliers due to the form deprivation paradigm. As shown in Table 1, significant correlations were found not only between anterior chamber depth and posterior ocular components (vitreous chamber depth and retinal thickness), but also between corneal parameters and posterior ocular dimensions (e.g., corneal J45 astigmatic component with vitreous chamber depth and retinal thickness). These results support the hypothesis that eye shape remodeling in the highly myopic eyeballs involves changes in both the anterior and posterior eye segments.

An important finding in this study is that the mRNA expression levels of *MMP2* and *TIMP2* genes were higher in form-deprived eyes than in normal eyes in sclera as a whole (average data), as well as in all three genes at the superior scleral region specifically (Fig. 3). As summarized in Supplementary Table S1, the three genes have shown differential expression patterns in sclera during the development of ametropia in previous studies. It should be noted that, unlike several studies which performed analyses on individual scleral layers (i.e., fibrous and/or cartilaginous) in chicks, we measured the mRNA expression level of the full-thickness scleral tissue. There is evidence that the molecular changes are opposite in the two scleral layers of chicks but the changes in fibrous sclera in chicks resemble those in tree shrew^43, 46^. Nevertheless, in agreement with our results, *MMP2* mRNA level in sclera have previously been found to increase in the treated eyes of form-deprived chicks^43^, in both treated and fellow eyes of chicks after 4 hours of monocular −7D lens wear^45^, and in the treated eyes of tree shrews after either 11 days of form deprivation^47^ or 4 days of −5D lens wear^18^. Compared to *MMP2*, expression levels of *TIMP2* and *TGFB2* in myopic sclera have shown inconsistent results across the different studies in the literature (Supplementary Table S1): *TIMP2* levels were not altered at multiple early time points after wearing +7D or −7D lenses^45^, but were found to decrease significantly after 10 days of form deprivation in chicks^43^. On the other hand, although the cartilaginous scleral *TGFB2* mRNA levels were found to increase after wearing +7D lens for 24 hrs in chicks^45^, the scleral *TGFB2* levels decreased after up to five days of form deprivation^17, 39, 48^ or −5D lens wear^17, 48^ in tree shrews (see Supplementary Table S1 for details). In short, the higher *MMP2* expression in myopic sclera was consistently found in the current and previous studies, regardless of the differences in experimental protocol and animal model. On the other hand, the patterns of change in *TIMP2* and *TGFB2* varied across studies. Nevertheless, the fact that differential mRNA levels were found at scleral/corneal regions in myopic chicks (Fig. 3) and regional variations in scleral proteoglycan synthesis were found in humans^49^ and myopic chicks^50^ underscores the importance of understanding the role of local mechanisms in controlling corneal and scleral remodeling.

A second important and novel finding was the regional specificity of the correlations between gene expression levels and refractive/structural parameters, these being most pronounced in the superior sclera. For example, the two key indicators for myopia development, the vitreous chamber depth and spherical-equivalent refractive error, showed moderate correlations with the mRNA levels of *MMP2* and *TIMP2* genes in the superior scleral (Fig. 4). These region-specific differential gene expressions are in line with our recent findings that full-field form deprivation actually induced a bigger eye expansion superiorly than inferiorly near the posterior pole (up to 20° eccentricity), i.e., an asymmetric posterior eye shape^35^. These results suggest the potential involvement of molecular changes concentrated at the superior scleral region in modulating the structural changes that occur in eyes developing myopia and astigmatism (see Table 1): a thinner retina, a deeper anterior chamber, a steeper corneal curvature, and a higher J45 astigmatism. It is possible that this combination of structural and molecular changes led to the unique astigmatic axis repeatedly found in form-deprived chicks, i.e., an axis typically oriented near the 90° meridian^15^. In contrast to the significant correlations between the two J45 astigmatic components and mRNA expressions at the superior sclera, only weak correlations were found between the astigmatic components and mRNA expressions at the nasal and inferior corneal regions, suggesting a less important and/or passive role of differential corneal gene expressions in manipulating the characteristics of astigmatism, at least at the time point we tested in this study. In light of the significant changes in mRNA expression concentrated at the superior sclera and the potential roles of the three genes in regulating eye shape remodeling, further studies are strongly in need for understanding the role of local, region-specific mechanisms during myopia development.

Active scleral remodeling has been shown to occur in a range of myopia models^22, 51^. Evidences from time-course experiments at the structural^52^ and molecular levels^19, 53^ indicate a more rapid, dramatic change during the early phase of myopic eye growth. In addition, recent studies in tree shrews^53^ and chicks^45^ have also shown that mRNA expression levels related to collagen fibril reorganization are time-dependent. In the current study, significantly higher expression of *MMP2*, *TIMP2*, and *TGFB2* mRNAs were found in the superior sclera of highly myopic-astigmatic eyes after 1 week of form deprivation in chicks, suggesting that local scleral structural remodeling was still in progress at this time point. However, given the potential differences in the mechanisms underlying form-deprivation and lens-induced myopia^23^, whether similar results would be found in highly myopic-astigmatic eyes induced through a closed loop condition (e.g., induced by wearing negative lens or sphero-cylindrical lens) awaits further studies.

Overall, our results demonstrated the structural and regional molecular changes in highly myopic-astigmatic eyes. It should be noted that the biometry and molecular changes were expressed as inter-ocular differences. The limitation of this approach is the potential interocular effects on the fellow untreated eyes of treated birds. However, given that no significant differences were found in the refractive parameters (refractive and corneal), axial structural parameters, or regional C_T_ values between the fellow untreated (left) eyes of the treated birds and the left eyes of the normal birds, these indicate that form deprivation did not produce significant effects on the fellow untreated eyes. The second limitation of this study is the use of full-thickness scleral tissue for molecular analysis. Although on average the form-deprived sclera showed higher expressions of *MMP2* and *TIMP2* than normal sclera, it remains unresolved if these changes were contributed by one or both scleral layers in chicks at this time point. Lastly, because the primary goal of this study was to test the hypothesis of whether genes known to participate in scleral structural remodeling show region-specific expressions in eyeball with abnormal anterior and posterior eye shapes, we only included chicks that developed high myopia with significant astigmatism. Further works are needed to confirm if similar region-specific expressions also occur in eyes with different refractive errors.

In conclusion, highly myopic and astigmatic eyes induced by monocular form deprivation not only exhibited elongated eyeball in both anterior and posterior segments, but also showed higher mRNA expressions of genes at the posterior sclera. The high correlations between biometry parameters and expression levels at specific scleral region indicate that local mechanism may manipulate the eye shape remodeling. Further studies are needed to confirm if these mechanisms found mostly in sclera are unique only to eyes with high myopia and astigmatism.

## Materials and Methods

### Form-deprivation treatment

Forty-seven White Leghorn (*Gallus Gallus domesticus*) chicks were used (treated, n = 39; normal, n = 8). Chicks were raised in the Centralized Animal Facilities of The Hong Kong Polytechnic University under a 12-hr light/12-hr dark cycle with free access to food and water. The average illuminance was ~100 lux at the chick’s eye level and the room temperature was 25 °C. All experiments were conducted in accordance with the Association for Research in Vision and Ophthalmology (ARVO) statement for the use of animals in Ophthalmic and Vision Research. The experimental protocols were approved by the Department of Health, Government of the Hong Kong Special Administrative Region (10–28 in DH/HA&P/8/2/4 Pt.3) and Animal Subject Experiment Subcommittee of The Hong Kong Polytechnic University (ASESC#0916).

To induce high myopia with astigmatism, chicks were reared with translucent occluders (diffusers) placed in front of their right eyes from post-hatch day 5 to day 12 (P5 - P12). At the end of the one-week treatment period, a modified Hartinger refractometer^54^ was used to identify birds that had developed high magnitudes of myopia (<−10D) and refractive astigmatism (>2D). Of the 39 treated birds, eight fulfilled these refractive-error criteria and additional biometric measures were taken to determine the corneal parameters and ocular axial dimensions by means of a custom-made corneal topographer^55^ and a high resolution (20 mHz) A-scan ultrasonography system, respectively^52^. Refractometry and A-scan ultrasonography were carried out while birds were anaesthetized (isoflurane inhalation, 1.0% to 1.5% in oxygen) whereas corneal topography were measured in awake chicks^55^. The age-matched control group (referred to as ‘normal group’, n = 8) received no treatment, but underwent the same biometry measurements. Following the above measurements, chicks were sacrificed by carbon dioxide asphyxiation.

### Tissue preparation

**Figure 7:**
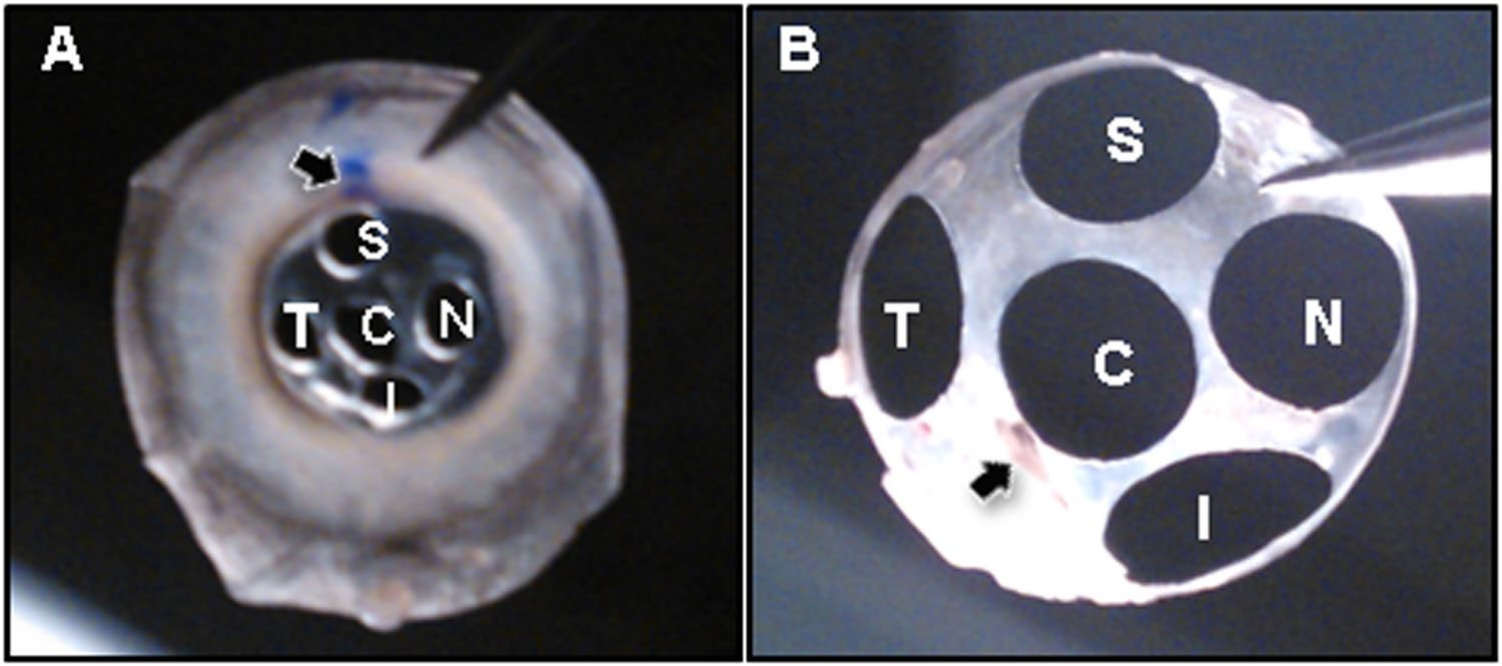
Corneal and scleral tissues used for mRNA analyses. Messenger RNA expressions of three genes were measured at different regions in chick’s cornea (**A**) and sclera (**B**). Arrows indicate the ink mark and pecten used to identify the orientations of cornea and sclera respectively. C, central; T, temporal; N, nasal; S, superior; I, inferior.

Immediately after the bird was sacrificed, eyes were enucleated carefully. To minimize potential confounding effects on RNA integrity due to the sequence of tissue preparation, the treated (or right eye) and fellow untreated eyes (or left eye) were enucleated in random sequence in different birds. The muscles and connective tissues remaining on the enucleated eye were removed, eyes were bisected with a razor blade along the equator of the eyeball and the vitreous body was removed. The remaining tissue was placed in ice-chilled 1% phosphate-buffered saline. The retina, lens and uvea were removed and discarded, and punches from the cornea and sclera were collected, using 1.5 mm- and 5 mm-diameter disposable trephines respectively, from 5 regions. These regions were chosen based on our previous findings that form deprivation induced both asymmetric posterior eye shape and significant amounts of astigmatism with axis oriented near 90° axis^35, 54^. As illustrated in Fig. 7, a mark made at the 12 o’clock conjunctiva was used as a reference to collect corneal tissues near the limbus (Fig. 7A), whereas the pecten was used as a landmark when collecting scleral tissues (Fig. 7B). The tissue punches were stored in 1.5 ml centrifuge tubes, snap-frozen in liquid nitrogen and stored at −80 °C until RNA was extracted.

### RNA quality check, RNA purification, and cDNA synthesis

The RNA quality of samples (0.5 μg) collected with and without using RNAlater was checked in a preliminary study using 1% denaturing agarose gels in formaldehyde containing running buffer (1xMOPS and formaldehyde). Both central corneal and scleral punches at the posterior pole were collected separately from a normal and a treated eye (separately from two birds) using 5 mm-diameter disposable trephines. The isolated tissues were further divided into 2 parts: one half was treated with RNAlater and the other half was not. As shown in the electrophoresis patterns in Supplementary Fig. 1, both bands of ribosomal RNAs (18S and 28S) were clearly visible and RNA qualities were comparable between the samples treated with or without RNAlater. Based on this result, all tissue samples in this study were collected without using RNAlater.

Total RNA from both tissue punches was purified with the RNeasy Fibrous Tissue Mini Kit (Qiagen, Utraco Greentech, Singapore) according to the manufacturer’s instructions. Tissues were dispersed and homogenized at a speed of 1600 rpm by a freezer mill (Mikro-Dismembrator, B. Braun Biotech, Melsungen, Germany). All samples were treated with DNase I according to the supplier’s instructions. The purity of RNA was determined by Nanodrop ND 1000 (NanoDrop Technologies), the optical density ratio (OD260/OD280) was between 1.8 and 2.1. All RNA samples were diluted to a concentration of 10 ng/μl in distilled water. Each RNA sample (50 ng) was reverse-transcribed to cDNA by using high capacity RNA to cDNA master mix (Life technologies, Tubas, Singapore) according to the manufacturer’s instructions. The cDNA reverse transcription reactions were prepared by mixing 10 μl of 2xRT buffer, 1μl of 20xRT enzyme mix, 5μl of RNA sample, and 4μl of nuclease-free water. Reverse transcription was carried out at 37 °C for 60 min, and halted by heating the sample to 95 °C for 5 min. Paired samples from right and left eyes were reverse-transcribed together to minimize batch effects.

### RT-PCR (reverse transcription –polymerase chain reaction) and sequencing

Primers for conventional RT-PCR were designed using Primer 3 Plus software (http://www.bioinformatics.nl/primer3plus; Life technologies, Hong Kong, China). Primers were designed to amplify a product size of 200–600 bp with GC content between 40% and 55%, and Tm values between 55 °C and 60 °C. To avoid the amplification of genomic DNA, the primers were designed to cross at least one exon junction for the specific amplification of cDNA. All primers were located within the coding region of the target genes. The detailed information of primer is shown in Supplementary Table S2. PCR was performed by using Hotstart Taq PCR master mix (QIAGEN, Utraco Greentech, Singapore) with primers specific for chick *MMP2*, *TIMP2*, and *TGFB2*. Both chick glyceraldehyde 3-phosphate dehydrogenase (*GAPDH*) and 18S ribosomal RNA (*RN18S*) were used as the reference gene for this step. In some samples the template was omitted as a negative control.

PCR was run under the following conditions: The initial denaturation was started at 95 °C for 5 min and followed by 32 cycles for *GAPDH* and *RN18S* genes, and 35 cycles for *MMP2*, *TIMP2* and *TGFB2* genes. Each cycle consisted of denaturation at 94 °C for 30 sec, annealing at 60 °C for 30 sec and extension at 72 °C for 30 sec, and a final extension step was performed at 72°C for 10 min.

PCR products were verified by DNA sequencing (Dragon technology Ltd., Hong Kong). Data were sent back and analyzed by a special Chromas software (provided by the Dragon technology Ltd., Hong Kong) and BLAST available from NCBI. All genes had 99% matching identities from BLAST.

### Real-time RT-PCR

Messenger RNA sequences for chicken *MMP2*, *TIMP2*, *TGFB2* and *RN18S* (accession numbers: *MMP2*, U07775; *TIMP2*, AF004664; *TGFB2*, X59080; and *RN18S*, AF173612) were obtained from the European Molecular Biology Laboratory sequence database (EMBL, Heidelberg, Germany), identical to those used in a previous chicks study^45^. The Taqman® qPCR gene expression assays used in this study were designed and the primers sequences for *MMP2*, *TIMP2*, *TGFB2* and *RN18S* are shown in Supplementary Table S3. Quantitative real-time PCR was performed in 96-well plates on an ABI 7500HT Real–Time PCR System (Applied Biosystems, New York, US). A total reaction volume of 20μl contained 10μl of 2 × TaqMan Universal PCR Master Mix (without uracil-N–glycosylase), 7μl of sterile water, 2μl of cDNA template, and 1μl of 20x Gene Expression Assay mix (including the primers and the marked probes). The thermal cycling conditions were: 95 °C for 10 min, followed by 40 cycles of 95 °C for 15 sec and 60 °C for 1 min. Samples were run in triplicate. One negative (i.e., no-template) control sample was included in each plate. In order to confirm the size of amplicons, *MMP2*, *TIMP2*, *TGFB2* and *RN18S* qPCR products were separated by electrophoresis on a 3% agarose gel with a 100 bp DNA ladder (Thermo Fisher Scientific, USA), and visualized using 1x Gel Red (Biotium, Hayward, CA, USA). The result shown in Supplementary Fig. 2 indicated that the primers (Supplementary Table S3) in TaqMan assays were specific and only one qPCR product was identified in the agarose gel.

The stability of *GAPDH* and *RN18S* at different regions was tested by performing TaqMan assay qPCR for both corneal and scleral tissues collected from 2 birds (4 eyes). Analyses of C_T_ values indicated that while *RN18S* did not show significant variability in either cornea (SD = 0.88) or sclera (SD = 0.80), the variability of *GAPDH* in sclera (SD = 3.26) was much higher than those in cornea (SD = 1.11). These results are in agreement with a previous study showing larger variability in *GAPDH* mRNA levels in chick’s fibrous sclera but 18 SRNA did not show significant difference between lens-treated and control eyes^45^. As a consequence, we chose *RN18S* as our endogenous control gene in subsequent analyses.

### Real time RT-PCR Data Analysis

RT-PCR quantification was performed using C_T_ values. “C_T_” is defined as the PCR cycle at which the fluorescence signal crosses a threshold line, which occurs during the exponential phase of the amplification curve. The smaller the C_T_ value, the higher the expression level. Because comparing the refractive parameters (refractive and corneal), axial parameters, and C_T_ values between the fellow untreated (left) eyes of the treated birds and the left eyes of the normal birds showed no significant differences (independent t-tests, all p > 0.05), we calculated the inter-ocular difference in gene expression (ΔΔC_T_) between the fellow eyes (i.e., treated and untreated fellow eyes in treated group, and right and left eyes in normal group) to compare the effects of visual manipulation on gene expressions between the treated and normal groups. Thus, at each of the five regions for each bird, the ΔΔC_T_ in gene expression between the treated (or right eye in normal group) and fellow eyes (or left eye in normal group) was calculated^56, 57^ with the equation:

$$\begin{array}{c}{{\rm{\Delta }}{\rm{\Delta }}C}_{{\rm{T}}}=[({{\rm{C}}}_{{\rm{T}}}\,{\rm{target}}\,{\rm{gene}}\,-\,{{\rm{C}}}_{{\rm{T}}}\,RN18S\,{\rm{gene}}){\rm{of}}\,{\rm{treated}}\,{\rm{eye}}\\ \quad \quad \quad \,\,\,-({{\rm{C}}}_{{\rm{T}}}\,{\rm{target}}\,{\rm{gene}}\,-\,{{\rm{C}}}_{{\rm{T}}}RN18S\,{\rm{gene}}){\rm{of}}\,{\rm{fellow}}\,{\rm{eye}}]\end{array}$$

The geometric mean of the triplicate samples for each bird was used for statistical analyses. To illustrate the expression levels in figure (Fig. 3), fold-change values above 1 (corresponding to gene expression that was greater in the treated eye sample compared to the fellow eye sample) was further expressed as 2^−ΔΔCT^, whereas fold-changes below 1 were expressed as −1/2^−ΔΔCT^.

### Statistical analyses

Refractive errors and corneal curvature (CC) were represented as spherical-equivalent (SE for refraction and CC for average corneal curvature), JO (=−0.5*C*cos2α) and J45 (=−0.5*C*sin2α) astigmatic components using power vectors, where C is the magnitude of the cylindrical power and α is the axis of the minus cylinder correcting lens^58^. To correlate with inter-ocular difference in gene expressions (ΔΔC_T_), effects of form deprivation on refractive errors and ocular dimensions were expressed as inter-ocular differences (treated/right eye minus fellow untreated/left eye). Paired *t*-tests were used to compare parameters between treated and fellow eyes. Independent *t*-tests or Mann-Whitney tests were used to test the difference in refractive and ocular dimensions between groups, depending on whether the within-group distribution was normally distributed. Spearman’s correlations were used to examine monotonic relationships between variables. Correction method such as Bonferroni’s method was not applied for multiple comparisons because the 3 genes and the biometric parameters could have inter-dependent effects (see results above^59^). All statistical tests were performed using Minitab 15.1.30.0 (Minitab Inc., USA) or SPSS Statistics 23.0 (IBM, USA) with a significance level set at α < 0.05.

## Electronic supplementary material

Supplementary Figure 1

Supplementary Figure 2

Supplementary Table S1

Supplementary Table S2

Supplementary Table S3
