# Supplementary material for: Region-specific differential corneal and scleral mRNA expressions of MMP2, TIMP2, and TGFB2 in highly myopic-astigmatic chicks
Source: Sci Rep. 2017 Sep 12;7:11423. doi: 10.1038/s41598-017-08765-6 (PMC5595952; doi:10.1038/s41598-017-08765-6)
Supplement: Supplementary file 3 — Supplementary Table S1 [file 41598_2017_8765_MOESM3_ESM.pdf]

Region-specific differential corneal and scleral mRNA expressions of *MMP2*, *TIMP2*, and *TGFB2* in highly myopic-astigmatic chicks.

Lisa Yan-yan XI[1], Shea Ping YIP[2], Sze Wan SHAN[1], Jody SUMMERS-RADA[3], *Chea-su KEE[1,4]

[1]School of Optometry, The Hong Kong Polytechnic University. Hung Hom, Kowloon, Hong Kong SAR.

[2]Department of Health Technology and Informatics, The Hong Kong Polytechnic University. Hung Hom, Kowloon, Hong Kong SAR.

[3]Department of Cell Biology, University of Oklahoma Health Sciences Center, Oklahoma City, OK, United States

[4]Interdisciplinary Division of Biomedical Engineering, The Hong Kong Polytechnic University. Hung Hom, Kowloon, Hong Kong SAR.

*Corresponding author:

Dr. Chea-su Kee PhD (Hong Kong)

School of Optometry, The Hong Kong Polytechnic University, Hong Kong

Tel: (852) 2766 7941 Fax: (852) 2764 6051 E-mail: c.kee@polyu.edu.hk

Authors	Animal	Tissue	Method	Starting age	Treatment and Duration	Optical/ Structural measures	Key findings
Schippert et al., 2006[45]	Male White Leghorn chicks	Entire fibrous and Cartilaginous sclera	Real-time PCR	P8-P11	• +7D or -7D in one eye, plano lens in fellow eye; control group wore plano lenses in both eyes<br>• mRNA measured after 4h, 24h, or 72h	--	• in fibrous sclera, lens type did not produce significant interocular difference, but treatment duration affected *MMP2* and *TGFB2* levels in a non-monotonic fashion over time<br>• in cartilaginous sclera, +7D lens wear for ≥ 24h ↑ *TGFB2* levels in the treated eye and produced significantly higher *TGFB2* level when compared to –7D lens wear.
Rada & Brenza, 1995[42]	White Leghorn chicks	11mm diameter posterior scleral punch and equivalent mass of anterior scleral ring	Digestion of 3H-gelatin after incubation with sclera sample	P2	• 11d of FD in one eye, fellow eye served as control	--	Elevated latent *MMP2* activity in form-deprived posterior sclera
Rada et al., 1999[43]	White Leghorn chicks	Posterior fibrous sclera	Northern blot	P2	• 10d of FD in one eye, fellow eye served as control<br>• 10d of FD followed by 1d of recovery<br>• 7d of FD in right eye, after which the FD was switched to left eye for 3d (zymography)	--	• Gelatinase A ↑ 128% in FD eyes and ↓ 80% in recovering eyes<br>• *TIMP2* ↓ 53% in FD eyes; no change in the recovering eye
Seko, Tanaka, & Tokoro, 1995[38]	Chick embryo	Entire sclera divided into fibrous and cartilaginous layers	Cell counts of chondrocyte and fibroblast	E17	Multiple growth factors were added in-vitro	--	• *TGFB* induced cell proliferation in a dose-dependent manner.
Seko, Shimokawa & Tokoro, 1995[37]	White Leghorn Chicks	8.5mm diameter posterior (temporal) eye cup	ELISA immunoassay	P2	12d of FD in one eye, fellow eye served as control	--	• FD increased *TGFB2* content and concentration in sclera and retina-RPE-choroid layers
Kusakari, Sata, & Tokoro, 1997[60]	White Leghorn Chicks	Sclera	Histology, immunohistochemistry	P2	2w of FD	-20~-30D myopia induced	• Increased cell density at posterior sclera, outermost layer, in between cartilaginous sclera and outer fibrous layer<br>• Increased proliferation of chondrocytes and active synthesis of ECM found at posterior sclera
Guggenheim & McBrien, 1996[16]	Tree Shrew	7mm diameter posterior sclera and the remaining equatorial sclera	SDS-PAGE, zymography	15d after eye opening	• 5d of FD, or 5d of FD followed by 3d of recovery, fellow untreated eye and eyes of control animals were also measured	FD induced relative myopia and axial elongation in treated eyes	• FD significantly increased active *MMP2* at posterior and equatorial sclera compared to fellow eyes<br>• active-to-latent *MMP2* ratio in FD group was significantly higher than all other groups.
Jobling et al., 2004[39]	Tree Shrew	7mm diameter posterior sclera	Quantitative real-time PRC	15d after eye opening	1d or 5d of FD, fellow eye served as control	--	• *TGFB2* levels decreased after 1d or 5d of FD<br>• *TGFB2* was the most potent isoform for the control of collagen synthesis
Siegwart & Norton, 2001[47]	Tree Shrew	Entire sclera	Quantitative competitive RT-PCR	24±1d after eye opening	• 11d of FD, fellow eye served as control<br>• 11d of FD followed by 4d of recovery	FD induced relative myopia and axial elongation when compared to fellow eye	• *MMP2* ↑ 66%; collagen mRNA ↓ 34% after 11d of MD<br>• *MMP2* ↓ 20%; collagen mRNA ↑ 33%; *TIMP1* ↑ 43% after 4days of recovery
Siegwart & Norton, 2005[18]	Tree Shrew	Entire sclera	Quantitative competitive RT-PCR	24±1d after eye opening	• 1, 2, 4, or 11d of -5D;<br>• 11d of -5D followed by 2 or 4d of recovery	Significant changes in refractive status and vitreous chamber depth	• *MMP2* ↑ 36% after 4d of -5D<br>• *MMP2* ↓ 28% after 11d of -5D followed by 4d of recovery
Gao et al., 2011[17]	Tree Shrew	Entire sclera	Quantitative real-time PCR	24±1d after eye opening	• 1, 4, or 11d of -5D;<br>• 11d of -5D followed by 1 or 4d of recovery	-5D lens wear induced myopia and removal of -5D lens wear induced hyperopic shift	• *TGFB2* levels decreased after 4d or 5d of -5D<br>• *TGFB2* levels decreased after 1d and increased after 4d of recovery
Guo et al., 2013[48]	Tree Shrew	Entire sclera	Quantitative real-time PCR	24±1d after eye opening	• 2 or 4d of -5D or FD;<br>• 11d of continuous darkness started from 17 after eyes opened	All treatments produced significant myopic shift, the magnitudes varied with duration of treatment	• *TGFB2* levels decreased after 2d or 4d of -5D or FD<br>• No significant effects on both *MMP2* and *TIMP2* levels

FD, form deprivation; h, hour; d, day; w, week; P, post-hatch day; Gp, group; ↑, increase; ↓, decrease

Supplementary Table S1. Previous studies using different myopia animal models for understanding the molecular expressions of MMP2, TIMP2, and TGFB2 in sclera.
