# Supplementary material for: Region-specific differential corneal and scleral mRNA expressions of MMP2, TIMP2, and TGFB2 in highly myopic-astigmatic chicks
Source: Sci Rep. 2017 Sep 12;7:11423. doi: 10.1038/s41598-017-08765-6 (PMC5595952; doi:10.1038/s41598-017-08765-6)
Supplement: Supplementary file 4 — Supplementary Table S2 [file 41598_2017_8765_MOESM4_ESM.pdf]

Region-specific differential corneal and scleral mRNA expressions of *MMP2*, *TIMP2*, and *TGFB2* in highly myopic-astigmatic chicks.

Lisa Yan-yan XI[1], Shea Ping YIP[2], Sze Wan SHAN[1], Jody SUMMERS-RADA[3], *Chea-su KEE[1,4]

[1]School of Optometry, The Hong Kong Polytechnic University. Hung Hom, Kowloon, Hong Kong SAR.
[2]Department of Health Technology and Informatics, The Hong Kong Polytechnic University. Hung Hom, Kowloon, Hong Kong SAR.
[3]Department of Cell Biology, University of Oklahoma Health Sciences Center, Oklahoma City, OK, United States
[4]Interdisciplinary Division of Biomedical Engineering, The Hong Kong Polytechnic University. Hung Hom, Kowloon, Hong Kong SAR.

*Corresponding author:

Dr. Chea-su Kee PhD (Hong Kong)
School of Optometry, The Hong Kong Polytechnic University, Hong Kong
Tel: (852) 2766 7941 Fax: (852) 2764 6051 E-mail: c.kee@polyu.edu.hk

Gene	Primer name	Primer sequence	Product length	Annealing temperature	GC%
GAPDH	forward	5'-CTAAGGCTGTGGGGAAAGTC-3'	535bp	55°C	55%
	reverse	5'-TCTATCAGCCTCTCCCACCT-3'	535bp	55°C	55%
RN18S	forward	5'-AAACGGCTACCACATCCAAG-3'	458bp	60°C	50%
	reverse	5'-CGTTTCCGAAAACCAACAAA-3'	458bp	60°C	40%
MMP2	forward	5'-TGGTGTTCCACAACCAAAGA-3'	435bp	60°C	45%
	reverse	5'-GGCAGCAACCAGAAGAGAC-3'	435bp	60°C	55%
TIMP2	forward	5'-GATGGCAAGATGCACATCAC-3'	373bp	60°C	50%
	reverse	5'-GGCGTGGACCAGTCTAACAT-3'	373bp	60°C	55%
TGFB2	forward	5'-GGTTGATCACTGCCTTCCATT-3'	482bp	60°C	50%
	reverse	5'-AGGGATGCTCTCGCACTTTA-3'	482bp	60°C	55%

Supplementary Table S2. RT-PCR primer information.
