# Supplementary material for: Region-specific differential corneal and scleral mRNA expressions of MMP2, TIMP2, and TGFB2 in highly myopic-astigmatic chicks
Source: Sci Rep. 2017 Sep 12;7:11423. doi: 10.1038/s41598-017-08765-6 (PMC5595952; doi:10.1038/s41598-017-08765-6)
Supplement: Supplementary file 5 — Supplementary Table S3 [file 41598_2017_8765_MOESM5_ESM.pdf]

Region-specific differential corneal and scleral mRNA expressions of *MMP2*, *TIMP2*, and *TGFB2* in highly myopic-astigmatic chicks.

Lisa Yan-yan XI[1], Shea Ping YIP[2], Sze Wan SHAN[1], Jody SUMMERS-RADA[3], *Chea-su KEE[1,4]

[1]School of Optometry, The Hong Kong Polytechnic University. Hung Hom, Kowloon, Hong Kong SAR.
[2]Department of Health Technology and Informatics, The Hong Kong Polytechnic University. Hung Hom, Kowloon, Hong Kong SAR.
[3]Department of Cell Biology, University of Oklahoma Health Sciences Center, Oklahoma City, OK, United States
[4]Interdisciplinary Division of Biomedical Engineering, The Hong Kong Polytechnic University. Hung Hom, Kowloon, Hong Kong SAR.

*Corresponding author:

Dr. Chea-su Kee PhD (Hong Kong)
School of Optometry, The Hong Kong Polytechnic University, Hong Kong
Tel: (852) 2766 7941 Fax: (852) 2764 6051 E-mail: c.kee@polyu.edu.hk

Target	Primer name	Primer sequence	Product length	Annealing Temperature	GC %
MMP2	Forward	5'-AGCTATGATGATGAC CGCAAGTG-3'	74bp	60°C	48%
	Reverse	5'-TCGTGGGCAGCA ACCAA-3'	74bp	60°C	59%
TGFB2	Forward	5'-CGTGCTCTAGATG CTGCCTATT-3'	60bp	60°C	50%
	Reverse	5'-TGGACGCAGGCAG CAATTAT-3'	60bp	60°C	50%
TIMP2	Forward	5'-CCTGCATCAAGAGG AGCGA-3'	78bp	60°C	58%
	Reverse	5'-CGATGTCGAGAAA CTCCTGCTT-3'	78bp	60°C	50%
18S	Forward	5'-GATCCATTGGAGG GCAAGTCT-3'	89bp	60°C	52%
	Reverse	5'-CGAGCTTTTTAACTG CAGCAACTTT-3'	89bp	60°C	40%

Supplementary Table S3. qPCR primers information.
